# An optimization solution of a laser plane in vision measurement with the distance object between global origin and calibration points

**DOI:** 10.1038/srep11928

**Published:** 2015-07-07

**Authors:** Guan Xu, Zhaobing Hao, Xiaotao Li, Jian Su, Huanping Liu, Lina Sun

**Affiliations:** 1 Traffic and Transportation College, Nanling Campus, Jilin University, Renmin Str. 5988#, Changchun, China; 2 Mechanical Science and Engineering College, Nanling Campus, Jilin University, Renmin Str. 5988#, Changchun, China

## Abstract

Equation construction of a laser plane demonstrates a remarkable importance for vision measurement systems based on the structured light. Here we create a simple 1D target with a cone at the bottom and a checkered pattern on the top to calibrate the equation of the laser plane in the view field of a camera. A group of 2D coordinates of the intersection points are extracted from the images with the 1D target at different positions. The objective function is constructed to optimize the coefficients of the laser plane by minimizing the difference between the distance from the feature point to the the origin point and the length of the 1D target. The projective lines of the optimized laser plane on the 3D calibration board overlap the real intersection lines in the experimental images. Finally, the comparison work about the influences of the non-Gaussian noise and point number is investigated experimentally. The experiments show that the method of the distance optimal object from the feature point to the origin point provides an accurate and robust calibration for the laser plane in structured light measurement.

Vision measurement based on structured light is one of the most important methods to reconstruct the surfaces of 3D objects, which improves 3D shape measurement with non-contact process, moderate speed, high reliability, and informative data^1^,^2^,^3^,^4^,^5^,^6^. Capitalizing on these capabilities, structured-light-based applications are being explored in measurement fields, such as inspection of mechanical parts, reconstruction of vehicle surface, and medical CAD/CAM^7^,^8^,^9^,^10^,^11^,^12^. A typical measurement system mainly includes a laser projector providing a laser plane on the object and a camera capturing the image with the bended laser line on the object^13^,^14^,^15^. The 2D coordinates of the laser line centers in the image are extracted and then transformed to 3D coordinates according to the calibration data of the laser plane. Therefore, it is the key point to precisely calibrate laser plane equation in the whole measurement task^16^,^17^. As the measurement precision relies on the calibration result of the laser plane, determining the accurate equation of a laser plane in the world coordinate system is meaningful to the vision measurement system adopting structured light.

A nonlinear measurement model is developed to the laser line image considering the radial distortion of the camera lens^18^. The way in which laser lines are extracted from the camera images is a natural formulation of the calibration problem as a nonlinear least squares problem. The nonlinear model reduces the errors in the generated 3D data by a precise camera model. A novel approach is proposed to generate the sufficient calibration points with high accuracy for structured light 3D vision^19^. The approach is based on a flexible 2D calibration target, composed of a photo-electrical aiming device, a 3D translation platform and an improved algorithm of back propagation neural network. The approach with an active laser beam for triangulation measurement is outlined for modeling and calibration^20^. The system works with the pattern of 2D beam-scanning illumination and one-dimensional slit-scanning detection with a photo-multiplier tube instead of a CCD camera. A procedure is proposed to calibrate a generic structured light system, including one camera and one projector^21^. The proposed procedure defines a unique coordinate system for both devices in the structured light system, and thus, a rigidity constraint is introduced into the transformation process. A method is proposed to easily determine all primitive parameters of a structured light vision sensor^22^,^23^. The technique requires the camera to observe a planar target shown at a few different orientations. A systematic method is explored for accurate and quick calibration of a 3D shape measurement system developed based on a structured light technique^24^. The key concept is to enable the projector to “capture” images like a camera, which makes the calibration of a projector the same as that of a camera. The calibration with corresponding system parameters, is proposed to effectively improve the measurement accuracy of 3D laser scanner for a large view depth^25^. A calibration object is able to move forwards and backwards precisely along the *z* direction of the world coordinate system. By planar fitting these points, the equation of the light stripe plane can be obtained. A technique is presented for intrinsic and extrinsic calibration of laser triangulation sensors integrated in a coordinate measuring machine^26^. This method performs calibration in a single step, which avoids the digitalization of a reference sphere in order to obtain the extrinsic parameters. An improved systematic calibration method is explained to enhance three key factors: calibration model, calibration artifact and calibration procedures^27^. The procedures calibrate the camera and projector simultaneously using the same reference points. A case study of radiometric calibration is presented for two phase-shift continuous wave terrestrial scanners^28^. Accordingly, it is important that the effects of distance and target reflectance should be carefully studied before using the intensity data from the terrestrial laser scanner. By using a criterion sphere, a calibration approach of a robot tool is introduced to calibrate the relation between the laser 3D scanner and the robot end-effector^29^. Meanwhile, by using the criterion sphere, an approach is provided to calibrate the pose of the robot relative to a turntable. A method is reported based on a novel algorithm^30^. The laser line position is obtained from the calibration algorithm, the high-speed CCD and the accurate determination of the laser marking location. The calibration method is built by the model of the geometrical relationship between the 3D coordinates of the laser stripe on the target and its digital coordinates in the image^31^. By this method, it is possible to calibrate the intrinsic parameters of the video system, the position of the image plane and the laser plane in a given frame in the same time. The calibration method with a 3D calibration board and a height gauge is proposed to calibrate the laser plane by the accurate known-position coordinates on the plane^32^. For producing the known-position coordinates, it is essential to move the height gauge on the horizontal plane of the 3D calibration board accurately. A simple method is presented for calibrating the laser plane by a 1D target with a mark on the top of the bar in the world coordinate system. The conical tip on the bottom of the target coincides with the origin point of the world coordinate system. The equation of the laser plane in the world coordinate system is calibrated by the optimal object of minimizing the difference of the real target length and the reconstructed target length with the laser plane equation. The position of the laser plane is determined by the distance object between the global origin of the world coordinate system and the calibration point on the end of the 1D target.

A flexible calibration method is explored in this paper, which reduces the cost of the calibration equipment and simplifies the calibration procedure. The rest of this paper is organized as follows: Section 2 presents the calibration process of laser plane in the vision measurement using structured light. The method is proposed for the calibration of the laser plane equation with a 1D target and the optimal distance object between the global origin and the feature point on the 1D target. Section 3 introduces the construction and solution procedures of the calibration model. Section 4 performs the calibration experiments and discusses the influencing factors for the precision of the calibration results. Section 5 summarizes this paper.

## Calibration Process

In the process of vision measurement based on structured light, we need to globally calibrate the laser plane equation to achieve the position and posture of laser plane in the world coordinate system. A method is proposed for the calibration of the laser plane equation with a 1D target. The bottom of the 1D target is located at the origin point of the 3D calibration board , the top of the 1D target is free and marked by a checkered pattern. Firstly, the 1D target is arbitrarily placed in the view filed of the camera. The bottom of the 1D target coincides with the origin point of the world coordinate system.The laser plane passes through the center of checkered pattern on the top of the target. A group of feature points are generated from the intersection points between the laser plane and the centers on the tops of the 1D target rod on different positions. Secondly, an optimal objective function is deduced to build the laser plane equation by the 2D coordinates of the feature points in the images and the transformation matrix of the camera calibration. Finally, the optimal coefficients are acquired according to the relationship between the coefficients of the laser plane equation and the value of the optimal objective function with multiple constraints. It is realized that the laser plane equation is solved by the optimization object of the distance from the origin point to a center of checkered pattern on the top of the 1D target in the world coordinate system.

**Figure 1:**
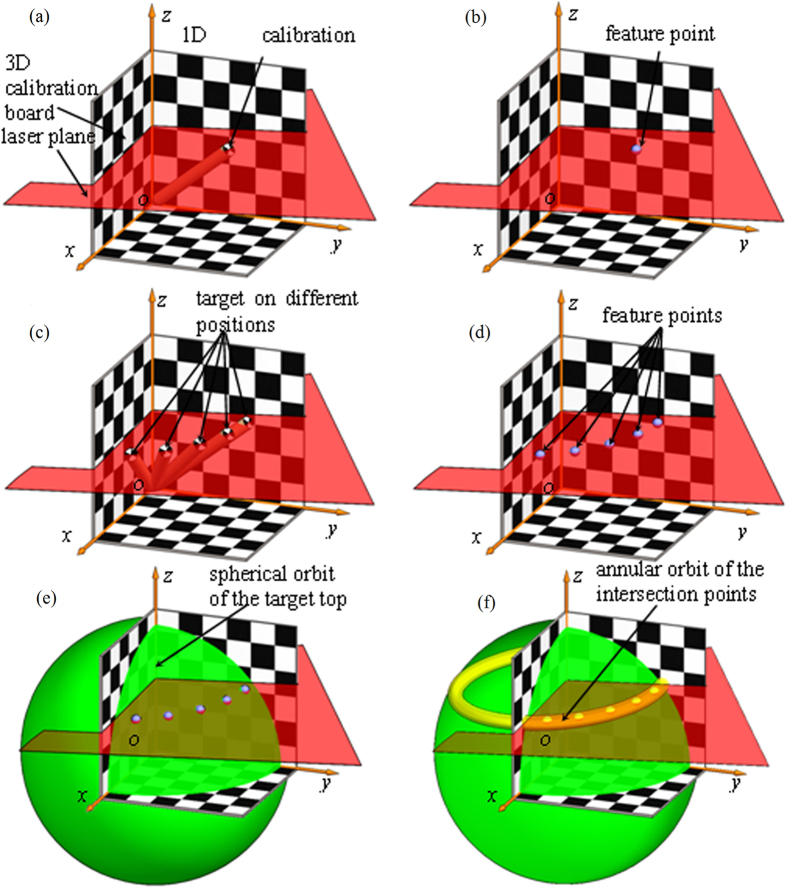
Calibration method of a laser plane using 1D target with a checkered pattern and a 3D calibration board. (**a**) 1D target position. The bottom of the 1D target rod is conical. The apex of the cone is positioned at the origin of the 3D calibration board. The top of the target is marked by a checkered pattern. (**b**) A feature point generated from the intersection point of the laser plane and the center of the checkered pattern on the top of the target. (**c**) The top of the 1D target moves to different positions where the laser plane intersects with the center of the checkered pattern. (**d**) A group of feature points are generated from the intersection points between the laser plane and the centers on the tops of the 1D target rod on different positions. (**e**) Spherical orbit of the 1D target top on different positions. (**f**) Annular orbit of the intersection feature points.

The 3D board is established in the camera calibration in Fig. 1. The coordinates of each corner point on the calibration board are fixed in the world coordinate system. We assume that the camera has no significant lens radial distortion or tangent distortion in this paper. The camera is modeled by the usual pinhole, which is represented as^33^
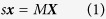
where***X*** = (*X*,*Y*,*Z*,1)’ are the world coordinates of the feature points on the 3D calibration board, ***x*** = (*x*,*y*,1)’ are the image coordinates of the corresponding feature points, *M* is a 3 × 4 transformation matrix, which shows the relationship between the 3D point ***X*** on the 3D calibration board and its 2D projection ***x*** in the camera image. *s* is a scaling factor. The elements of matrix *M* are worked out by the least square method.

The 1D target in Fig. 1a is adopted for the laser plane calibration. The bottom of the 1D target rod is conical. The apex of the cone is positioned at the origin of the 3D calibration board. The top of the 1D target rod is free and marked by a checkered pattern. The method for achieving the image coordinates of the feature points on the top of the 1D target is explained in Fig. 1b. The 1D target and the 3D calibration board are placed in front of the camera. The apex of the conical bottom of the 1D target is set at the origin point of the 3D calibration board.The laser plane passes through the center of the checkered pattern on the top of the 1D target by adjusting the position of the top of the 1D target in the view filed of the camera. Then the center of the checkered pattern on the top of the 1D target is the common point between the laser plane and the top of the 1D target. Furthermore, the length of the 1D target is a known quantity. *A*_*i*_ is the intersection point between the laser plane and the center of the checkered pattern. As the apex of the conical bottom of the 1D target coincides with the origin point of the 3D calibration board, the distance from the intersection point *A*_*i*_ to the origin point of the 3D calibration board is equal to the known length of the 1D target. The top of the 1D target moves to different positions where the laser plane intersects with the center of the checkered pattern . As a result, a group of feature points are obtained from the intersection points between the laser plane and the center of the checkered pattern as shown in Fig. 1c,d. Hence, as illustrated in Fig. 1e, the motion route of the feature points on the 1D target is a sphere that takes the length of the 1D target as the radius and the origin point as the center. The motion route of the intersection points between the laser plane and the center of the checkered pattern is an intersection circle between the sphere and the laser plane, as the yellow ring in Fig. 1f. Consequently, a couple of 2D coordinates are derived from the intersection point between the laser plane and the center of the checkered pattern in the corresponding image. A Group of 2D coordinates of the intersection points are provided by analyzing the images with the 1D target at different positions. According to the object that the distance from the feature point to the the origin point of the 3D calibration board is equal to the length of the 1D target, the laser plane equation in the world coordinate system is solved by optimizing the objective function.

## Construction and Solution of Calibration Model

The laser plane passing through all the feature points on the 1D target can be considered as an ideal plane. The general equation of a laser plane is given by Eq. (2):
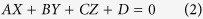
where (*X*_*i*_, *Y*_*i*_, *Z*_*i*_) are the 3D coordinates of the intersection points between the laser plane and the center of the checkered pattern on the top of the 1D target in the world coordinate system, *A*, *B*, *C*, *D* are the coefficients to be calibrated in the plane equation.

The expansion of Eq. (1) and the laser plane Eq. (2) are simultaneously given by
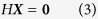
where 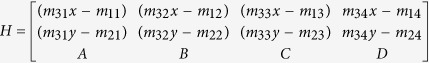, (*x*, *y*) are the 2D image coordinates of the intersection points between the laser stripe and the center of the checkered pattern on the top of the 1D target, *m*_*ij*_ is the *i*-th row and *j*-th column element in the transformation matrix *M*.

The 3D coordinates ***X*** of the intersection points in the world coordinate system are expressed by
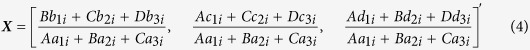
where (*a*_1*i*_, *a*_2*i*_, *a*_3*i*_, *b*_1*i*_, *b*_2*i*_, *b*_3*i*_, *c*_1*i*_, *c*_2*i*_, *c*_3*i*_, *d*_1*i*_, *d*_2*i*_, *d*_3*i*_)’ = *G**x***,
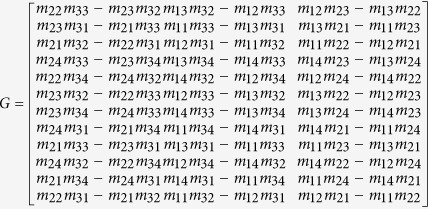


The distance from the center *A*_*i*_(*X*_*i*_, *Y*_*i*_, *Z*_*i*_) of the checkered pattern on the top of the 1D target to the origin point of the 3D calibration board is represented by the module of 3D coordinates ***X*** of the intersection points in the world coordinate system in Eq. (5):
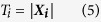
where *T*_*i*_ is the distance from the reconstructed coordinate *A*_*i*_(*X*_*i*_, *Y*_*i*_, *Z*_*i*_) to the origin point in the world coordinate system. *A*_*i*_(*X*_*i*_, *Y*_*i*_, *Z*_*i*_) is reconstructed by the 2D coordinates of the feature points in the images.

The length of the 1D target should be equal to the spatial distance from the origin point of the 3D calibration board to the intersection point between the laser plane and the center of the checkered pattern on the top of the 1D target. Therefore, the optimization goal is the minimum difference between the sum of squared length of the 1D target and the sum of squared spatial distance from the feature point to the origin point of the 3D calibration board. The feature point is the intersection point between the laser plane and the center of the checkered pattern on the top of the 1D target. The optimal objective function *f*(*A*, *B*, *C*, *D*) with constraints is given by Eq. (6). The aim of the optimal objective function *f*(*A*, *B*, *C*, *D*) is the minimum difference of the spatial distance and the length of the 1D target:
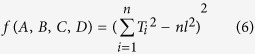
where *l* is the length of the 1D target. *A*, *B*, *C*, *D* are the coefficients of the laser plane equation. *n* is the number of the target images in the experiments. The spatial distance *T*_*i*_from the intersection point to the origin point calculated by the coordinates of the reconstructed intersection points is equal to the length *l* of the 1D target. i.e., the corresponding *A*, *B*, *C*, *D* are the coefficients of the laser plane equation on the condition that the value of the function *f*(*A*, *B*, *C*, *D*) is close to zero. As there are only three independent coefficients among the coefficients *A*, *B*, *C*, *D* in the laser plane equation. When *C* = 1 is defined, the optimal objective function can be solved with the coefficients *A*, *B*, *D*. The constraints of the variables *A*, *B*, *D* can be preliminarily determined by the light projection on the 3D calibration board. In order to minimize the spatial distance, the variable *D* varies in its scope firstly. Then we obtain a series of binary functions related to *A* and *B*. Each function has a minimal value that is solved by Matlab function *min* in the constraint scopes of the variables *A*, *B*. We consider the smallest one among the minimal values of the series of binary functions as the the minimum. Finally, the *A*, *B*, *D* corresponds to the minimum are the optimal values.

## Experiments and Discussions

The experimental system consists of a laser projector with 635 nm wave length, a camera (DH-HV3102UC-T) with 5 mm lens, a tripod, a 3D calibration board, a 1D target, and a computer with 3.4 GHz processor, 4CPUs and 6 GB RAM. The size of the 3D calibration board is 500 × 500 × 500 mm. 60 × 60 mm chessboard-grids cover the 3D calibration board. The length of the 1D target is 430 mm. The image resolution is 2048 × 1536.

For performing the global calibration of the laser plane, the relative positional relationship between the camera and the 3D calibration board should be determined firstly. The 3D calibration board is employed to calibrate the camera. The 3D calibration board is placed in front of the camera. The intersection point of the three planes of the 3D calibration board is considered as the origin point of the world coordinate system. 9 feature points are chosen on each plane of the 3D calibration board. The camera obtains the corresponding 2D coordinates of the 27 feature points on the 3D calibration board. The elements of the matrix *M* are calculated by the least square method. The transformation matrix *M* is obtained from two groups of experiments as illustrated in Table 1. The object can be measured after placing it in the view filed of the camera.

**Figure 2:**
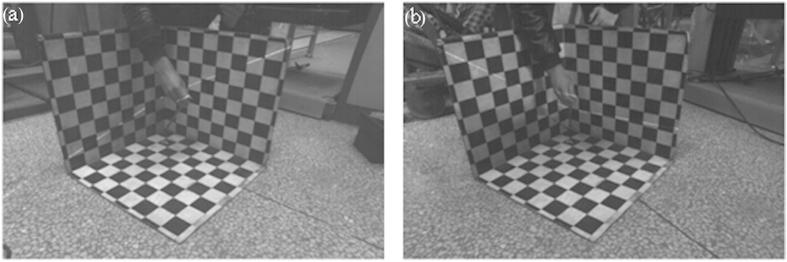
The calibration experiments of the laser plane using a 1D target with a checkered pattern. (**a**) A sample picture in the first group. (**b**) A sample picture in the second group.

**Figure 3:**
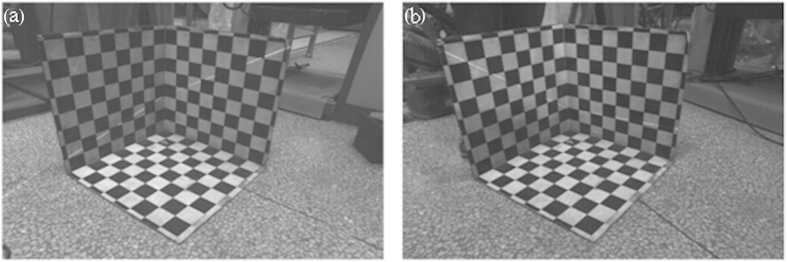
The crossing laser lines between the laser plane and the 3D calibration board. (**a**) A sample picture in the first group. (**b**) A sample picture in the second group.

The 1D target in Fig. 2 is utilized in the laser plane calibration. The intersection line between the laser plane and the checkered pattern passes through the center of the checkered pattern by adjusting the position of the top of the 1D target in measuring space. Two group experiments are performed. Each experiment captures the images of the 1D target at twenty different positions. The feature point coordinates are obtained from the images respectively. The optimal objective function is calculated with the independent coefficients *A*, *B*, *D* in the laser plane equation when *C* is equal to 1. The coefficients *A*, *B* stand for the slopes of the intersection lines between the laser plane and the 3D board respectively. The coefficient *D* indicates the intercept at *z* axis of the intersection lines. The initial equation coefficients can be determined by the positions of the intersection lines in Fig. 3. In the first laser plane equation, the initial value of coefficients are *A* = 0.25, *B* = −0.55, *C* = 1, *D* = −185, the constraint scopes of the variables are 0.21 < *A* < 0.29, −0.62 < *B* < 0.53, −200 < *D* < −170. In the second laser plane equation, the initial value of coefficients are *A* = −0.7, *B* = −0.05, *C* = 1, *D* = −115, the ranges of the variables are −0.77 < *A* < −0.67, −0.11 < *B* < −0.2, −130 < *D* < −100. Two optimal objective functions *f*_1_(*A*, *B*, *D*), *f*_2_(*A*, *B*, *D*) with constraints are constructed by Eq. (6).

The optimal objective functions are optimized for obtaining the laser plane equations which are indicated in Eqs. (7), (8):
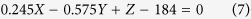

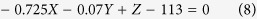


**Figure 4:**
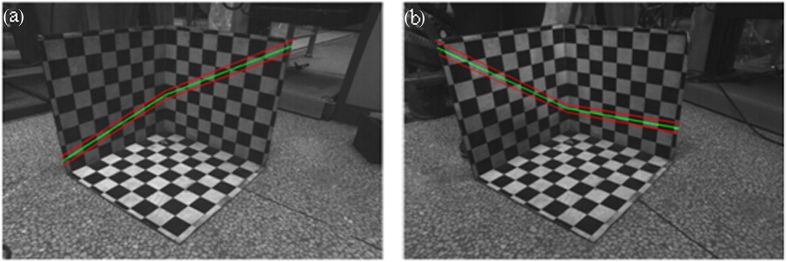
The projected laser lines according to the laser plane equation. The green lines are the reconstructed laser lines based on the solutions of the objective function. The red ones are the constraint scope of the objective function. (**a**) The projected laser lines in the first group. (**b**) The projected laser lines in the second group.

To test and verify the validity of the calibration results, two equations of laser planes are projected to the *xoz* plane and the *yoz* plane of the 3D calibration board. Then two green lines are generated in Fig. 4. The red lines in Fig. 4 represent the scope boundaries of the projective laser lines reconstructed by the constraint scopes of the optimal objective functions. Comparing the two reconstructed lines with the real intersection lines between the laser plane and the 3D calibration board, the green projective lines of the laser plane on 3D coordinate system coincides with the two real projective lines of the laser plane on the 3D calibration board. According to the results above, it is evident that the accurate calibration of the laser plane is achieved by the method of single point positioning of the center of the checkered pattern and the optimization object of the distance from the origin point to a center of checkered pattern on the top of the 1D target.

**Figure 5:**
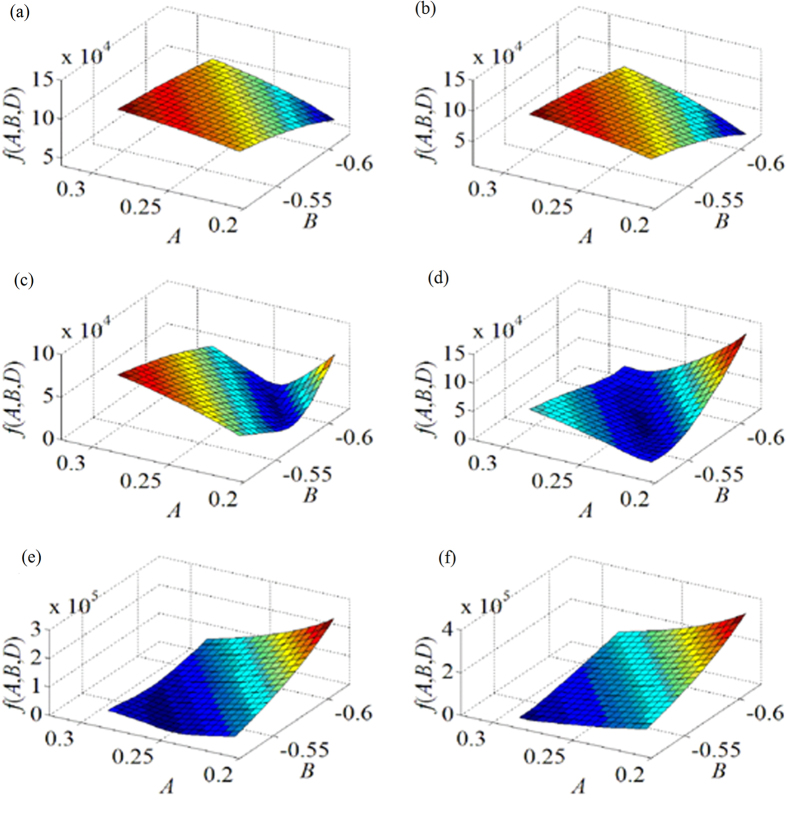
Relationship between the objective function *f*(*A*, *B*, *D*) and the coefficients *A*, *B* of the plane equation in the first group experiments. The variation of the objective function is observed from the subfigures with a decreasing variable *D*. (**a**) *D* = −155. (**b**) *D* = −165. (**c**) *D* = −175. (**d**) *D* = −184. (e) *D* = −195. (f) *D* = −205.

We further analyzed the experiment results according to the optimal objective function. The images plotted by the first optimal objective function *f*_1_(*A*, *B*, *D*) are shown in Fig. 5. As the objective function is a function with three variables, *A*, *B* are taken as the *x*, *y*-axis while *D* = −155 as the starting point with a 10 interval in Fig. 5. We discuss the value of the optimal objective function varies with the parameters *A*, *B*, *D*, respectively.

The scope of the extreme points and the minimal value regularly varies with the decrease of *D*. The extremum of the surface is not obvious on the condition that *D* = −155 and *D* = −165. The minimum of the surface is observed when the value of *D* is reduced from −175 to −195. When *D* = −175, *f*_1_(*A*, *B*, *D*) reaches its minimal value of 1.845 × 10^3^ in the scopes of 0.22 < *A* < 0.24, −0.62 < *B* < −0.59. When *D* = −184, *f*_1_(*A*, *B*, *D*) gains the minimal value of 1.130 × 10^3^ in the scopes of 0.24 < *A* < 0.26, −0.59 < *B*< −0.56. Furthermore, the minimum value of *f*_1_(*A*, *B*, *D*) diminishes with the reduction of *D*. When *D* = −195, *f*_1_(*A*, *B*, *D*) derives a minimal value of 2.1629 × 10^3^ in the scopes of 0.25 < *A* < 0.27, −0.55 < *B* < −0.53. The minimal value increases with the diminution of *D*. When *D* = −205, the extremum of the surface vanishes. The minimum value augments while *D* declines. As stated above, when *D* = −184, *f*_1_(*A*, *B*, *D*) reaches its minimal value. The coefficients *A*, *B* are in the scopes of 0.24 < *A* < 0.26, −0.59 < *B* < −0.56. *A* = 0.245, *B* = −0.575, *D* = −184 are the variables corresponding to the minimum value of the objective function *f*_1_(*A*, *B*, *D*). The variables of *A*, *B*, *D* here minimize the difference between the length of the 1D target and the distance from the feature point to the origin point. It agrees with the experiment results well.

The variation trends of *f*_1_(*A*, *B*, *D*) are different in the directions of *A* and *B* while *D* declines in Fig. 5. On the condition that *D* = −155 and *D* = −165, the value of *f*_1_(*A*, *B*, *D*) decreases when the value *A* is on the decline. The value of *f*_1_(*A*, *B*, *D*) diminishes in the direction of *B* while the value of *B* is reduced. In the direction of *A*, the value of *f*_1_(*A*, *B*, *D*) reduces firstly, and then enlarges when the value of *D* is dropped from −175 to −195. In the direction of *B*, the value of *f*_1_(*A*, *B*, *D*) lessens firstly, and then becomes larger with the diminution of *B*. When *D* = −205, the extremum of the surface vanishes. The value of *f*_1_(*A*, *B*, *D*) increases in both directions of *A* and *B* with the attenuation of *A* and *B*, respectively.

**Figure 6:**
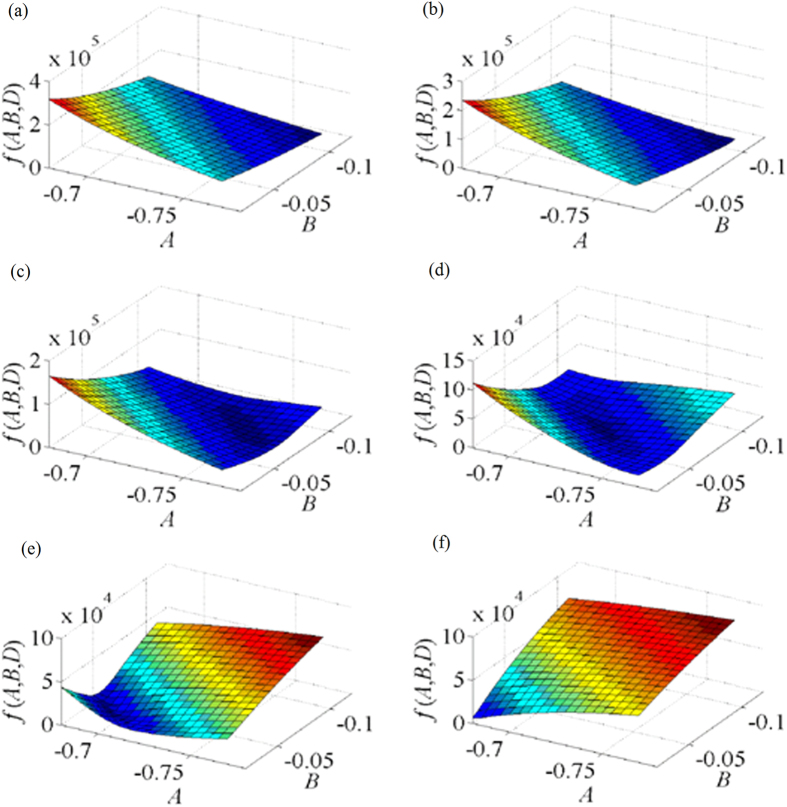
Relationship between the objective function *f*(*A*, *B*, *D*) and the coefficient s *A*, *B* of the plane equation in the second group experiments. The variation of the objective function is observed from the subfigures with a decreasing variable *D*. (**a**) *D* = −85. (**b**) *D* = −95. (**c**) *D* = −105. (**d**) *D* = −113. (**e**) *D* = −125. (f) *D* = −135.

The images of the second optimal objective function are illustrated in Fig. 6. The function takes *A*, *B* as the *x*, *y*-axis while *D* = −85 as the starting point with an interval of 10. We discuss the value of the optimal objective function varies with the parameters *A*, *B*, *D*, respectively.

The scope of the extreme points and the minimal value varies regularly with the reduction of *D*. The extreme point of the surface is not distinct on the condition that *D* = −85, *D* = −95. The minimums of the surface are evident when the value of *D* drops from −105 to −125. When *D* = −105, *f*_2_(*A*, *B*, *D*) reaches its minimal value of 1.236 × 10^3^ in the scopes of −0.76 < *A* < −0.74, −0.09 < *B* < −0.07. When *D* = −113, *f*_2_(*A*, *B*, *D*) gets the minimal value of 749.249 in the scopes of −0.74 < *A* < −0.71, −0.08 < *B* < −0.06. Meanwhile the minimum value of *f*_2_(*A*, *B*, *D*) decreases with the diminution of *D*. When *D* = −125, *f*_2_(*A*, *B*, *D*) develops a minimal value of 1.583 × 10^3^ in the scopes of −0.7 < *A* <−0.68, −0.06 < *B* < −0.04. The minimum value increases while *D* drops down. When *D* = −135, the extremum of the surface vanishes. As described above, when *D* = −113, *f*_2_(*A*, *B*, *D*) reaches its minimal value. The coefficients *A*, *B* are in the scopes of −0.74 < *A* < −0.71, −0.08 < *B* < −0.06. *A* = −0.725, *B* = −0.07, *D* = −113 are the variables corresponding to the minimum value of the objective function *f*_2_(*A*, *B*, *D*). The coefficients *A*, *B*, *D* are satisfied with the optimized goal that the length of the 1D target is equal to the distance from the feature point on the top of the 1D target to the origin point and accords with the experiment results.

The variation trends of *f*_2_(*A*, *B*, *D*) are different in the directions of *A* and *B* while *D* decreases. On the condition that *D* = −85 and *D* = −95, the value of *f*_2_(*A*, *B*, *D*) decreases when the value *A* is on the decline. The value of *f*_2_(*A*, *B*, *D*) diminishes in the direction of *B* while the value of *B* is reduced. In the direction of *A*, the value of *f*_2_(*A*, *B*, *D*) reduces firstly, and then enlarges when the value of *D* is dropped from −105 to −125. In the direction of *B*, the value of *f*_2_(*A*, *B*, *D*) decreases first, and then becomes larger with the diminution of *B*. When *D* = −135, the extremum of the surface vanishes. The value of *f*_2_(*A*, *B*, *D*) increases in both directions of *A* and *B* with the decline of *A* and *B*, respectively.

**Figure 7:**
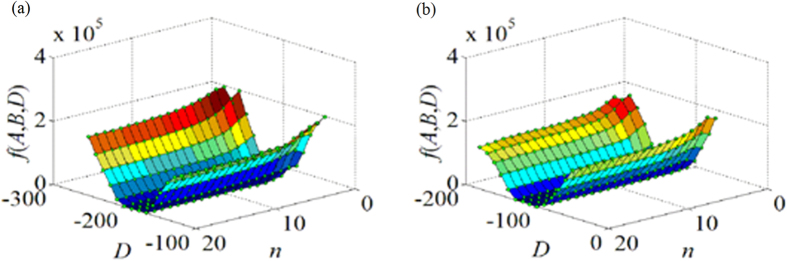
Relationship between the minimum of the objective function *f*(*A*, *B*, *D*), the equation coefficient *D* and the group number n. (**a**) The relationship in the first group. (**b**) The relationship in the second group.

Figure 7 shows the value variation of the objective function related to the laser plane coefficient *D* and the group number *n* of the 1D target experimental images. We can observe the value of the objective function varies with the change of the laser plane coefficient *D* and the group number *n* visually.

Figure 7a indicates the relationship between the value of *f*_1_(*A*, *B*, *D*), the laser plane coefficient *D* and the data group number *n* in the first experiment. In the direction of *D*, the value of *f*_1_(*A*, *B*, *D*) reduces at the beginning, and then enlarges when the value of *D* increases from −300 to −100. Along the direction of *D*, *f*_1_ (*A*, *B*, *D*) reaches its minimal value around *D* = −200. In the direction of *n*, the value of *f*_1_(*A*, *B*, *D*) gradually diminishes when the data group number *n* in the experiment enlarges from 0 to 20. If *n* is bigger than 15, the decrease trend for the value of *f*_1_(*A*, *B*, *D*) tends to flatten slowly. When *n* = 20 and *D* = −184, *f*_1_(*A*, *B*, *D*) gains its minimal value.

Figure 7b shows the relationship between the value of *f*_2_(*A*, *B*, *D*) , the laser plane coefficient *D* and the data group number *n* in the second experiment. In the direction of *D*, the value of *f*_1_(*A*, *B*, *D*) abates firstly, and then increases when the value of *D* augments from −200 to 0. When *n* = 20 and *D* = −113, *f*_1_(*A*, *B*, *D*) reaches its minimal value. In the direction of *n*, the value of *f*_1_(*A*, *B*, *D*) gradually reduces when the group number *n* enlarges from 0 to 20. The results of these two experiments indicate that the optimal objective function can obtain the stable optimal solutions when the number of data group is close to 20 in the experiment.

**Figure 8:**
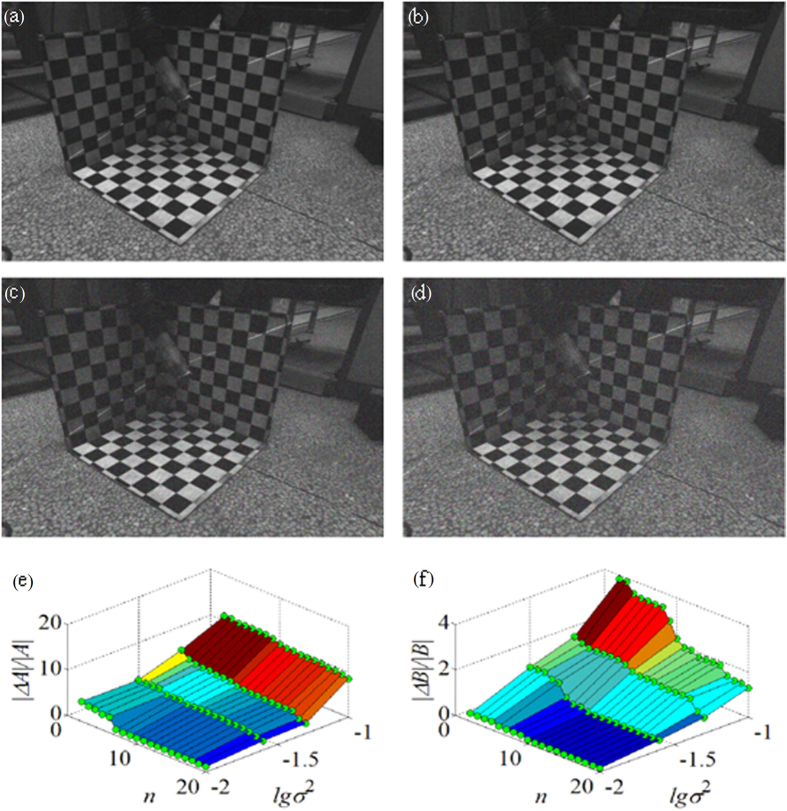
The analysis of noise influence on the first experiment results. The original images in the experiment are added with different Gaussian noise. (**a**) The variance *σ*^2^ of Gaussian noise is equal to 0.01. (**b**) The variance *σ*^2^ of Gaussian noise is equal to 0.02. (**c**) The variance *σ*^2^ of Gaussian noise is equal to 0.05. (**d**) The variance *σ*^2^ of Gaussian noise is equal to 0.1. (**e**) The relationship between the noise level lg*σ*^2^, the data group number *n* and the relative error of the laser plane coefficient |∆*A|/|A|*. (**f**) The relationship between the noise level lg*σ*^2^, the data group number *n* and the relative error of the laser plane coefficient |∆*B|/|B|*.

Gaussian noise is added to the experimental images to analyze the influence of the noise on the experiment results. The original images in the first experiment are added with different Gaussian noise of which the variance *σ*^2^ is equal to 0.01, 0.02, 0.05, 0.1, respectively, as shown in Fig. 8a–d. The relationship between the noise level, the data group number *n* and the relative error of the laser plane coefficients *A* and *B* are illustrated in Fig. 8e,f, respectively. It is more convenient to observe the noise data while Gaussian noise on the noise axis is expressed by the denary logarithm of the variance. In Fig. 8e,f, the relative errors of the laser plane coefficients *A* and *B* decrease when Gaussian noise is on the decline. In the direction of *n*, the relative errors of the laser plane coefficients *A* and *B* gradually diminish when the data group number *n* in the experiment enlarges from 0 to 20. The decrease trend tends to be smooth finally. As the coefficients *A*, *B* represent the slopes of the intersection lines between the laser plane and the 3D calibration board respectively. The slope of the intersection line between the laser plane and the *xoz* plane of the 3D calibration board is smaller than the slope of the intersection line between the laser plane and the *yoz* plane, i.e. |*A*| < |*B*|, as shown in Fig. 8a–d. The pixel offsets are generated when Gaussian noise is added to the images. The influence of the pixel offsets is identical when the noise with same intensity is added to the images. Therefore, the changes of the coefficients *A*, *B* are equal, i.e. Δ*A*=Δ*B.* Thus, |Δ*A*/*A*| > |Δ*B*/*B*|, the relative error of the laser plane coefficient *A* is larger than the relative error of the laser plane coefficient *B,* as depicted in Fig. 8e,f.

**Figure 9:**
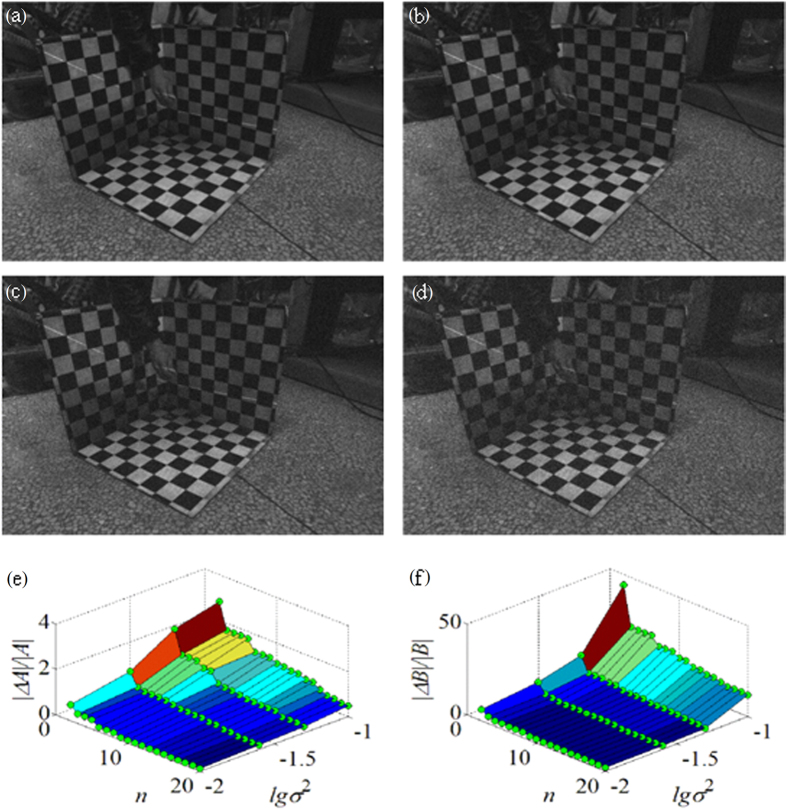
The analysis of noise influence on the second experiment results. The original images in the experiment are added with different Gaussian noise. (**a**) The variance *σ*^2^ of Gaussian noise is equal to 0.01. (**b**) The variance *σ*^2^ of Gaussian noise is equal to 0.02. (**c**) The variance *σ*^2^ of Gaussian noise is equal to 0.05. (**d**) The variance *σ*^2^ of Gaussian noise is equal to 0.1. (**e**) The relationship between the noise level lg*σ*^2^, the data group number *n* and the relative error of the laser plane coefficient |∆*A|/|A|*. (**f**) The relationship between the noise level lg*σ*^2^, the data group number *n* and the relative error of the laser plane coefficient |∆*B|/|B|*.

The original images in the second experiment are added with different Gaussian noise of which the variance *σ*^2^ is equal to 0.01, 0.02, 0.05, 0.1, respectively, as shown in Fig. 9a–d. Figure 9e,f show the relationships between the noise value, the data group number *n* and the relative error of the laser plane coefficients *A* and *B* , respectively. In order to observe the noise data with the same interval, Gaussian noise on the noise axis is represented by the denary logarithm of the variance. In Fig. 9e,f, the relative errors of the laser plane coefficients *A* and *B* reduce when Gaussian noise weakens. In the axis of *n*, the relative errors of the laser plane coefficients *A* and *B* gradually diminish when the data group number *n* in the experiment augments from 0 to 20. The decrease trend tends to be flat finally. As the coefficients *A*, *B* represent the slopes of the intersection lines between the laser plane and the 3D calibration board, the slope of the intersection line between the laser plane and the *xoz* plane of the 3D calibration board is larger than the slope of the intersection line between the laser plane and the *yoz* plane, i.e. |*A*| > |*B*|, as shown in Fig. 9a–d. The pixels offset when Gaussian noise is added to the images. The effects of the pixel offsets are identical when the noise with same intensity is added to the images. Therefore, the variation of coefficients *A*, *B* are equal, i.e. Δ*A*=Δ*B.*
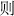
|Δ*A*/*A*| < |Δ*B*/*B*|, Thus, |Δ*A*/*A*| < |Δ*B*/*B*|, the relative error of the laser plane coefficient *A* is smaller than the relative error of the laser plane coefficient *B*, as depicted in Fig. 9e–f.

**Figure 10:**
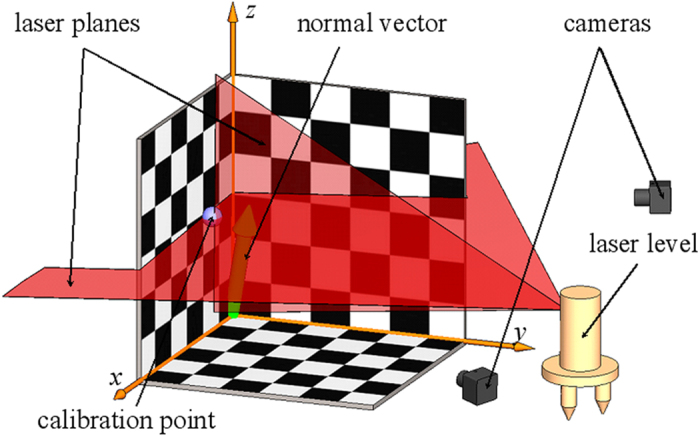
Hu’s calibration method. Two cameras and one laser level are placed in front of the 3D calibration board. The laser level generates two laser planes that are vertical to each other. Two crossing lines are generated from the laser planes on the 3D calibration board. The calibration point is the crossing point between two crossing lines. The 3D coordinates of the measurement point are reconstructed by the calibration results of two cameras.

We further evaluate our calibration method by comparing its experimental results with the results of the calibration method presented by Hu^34^. The experimental framework is conducted as shown in Fig. 10. Two cameras and one laser level are adopted in the experiment. The laser level generates two laser planes that are vertical to each other. The binocular cameras and the laser level are used to simulate the measurement performance of the laser range finder. The crossing line of the laser planes is considered as the measurement laser line of the laser range finder. The crossing point between the crossing line and the 3D calibration board refers to the measurement point of the laser range finder. The 3D coordinates of the measurement point are reconstructed by the calibration results of two cameras. Then the laser plane is calibrated by Hu’s method. As we attach the world coordinate system on the 3D board, the normal vector of the calibrated laser plane in the world coordinate system can be obtained by the calibration. The noise magnitude and calibration precision should be investigated by the experiments^35^. Three direction angles *α*, *β*, *γ* and three magnitude components *Dis*_*x*_, *Dis*_*y*_, *Dis*_*z*_ of the normal vector from the origin point to the laser plane are used as the calibration outputs. The real values of the laser plane are measured by a precise vernier caliper. The comparison work is divided in two aspects: one is to compare the noise effects of the two methods. The other one is to evaluate the effects of the number of the feature points from the two methods.

**Figure 11:**
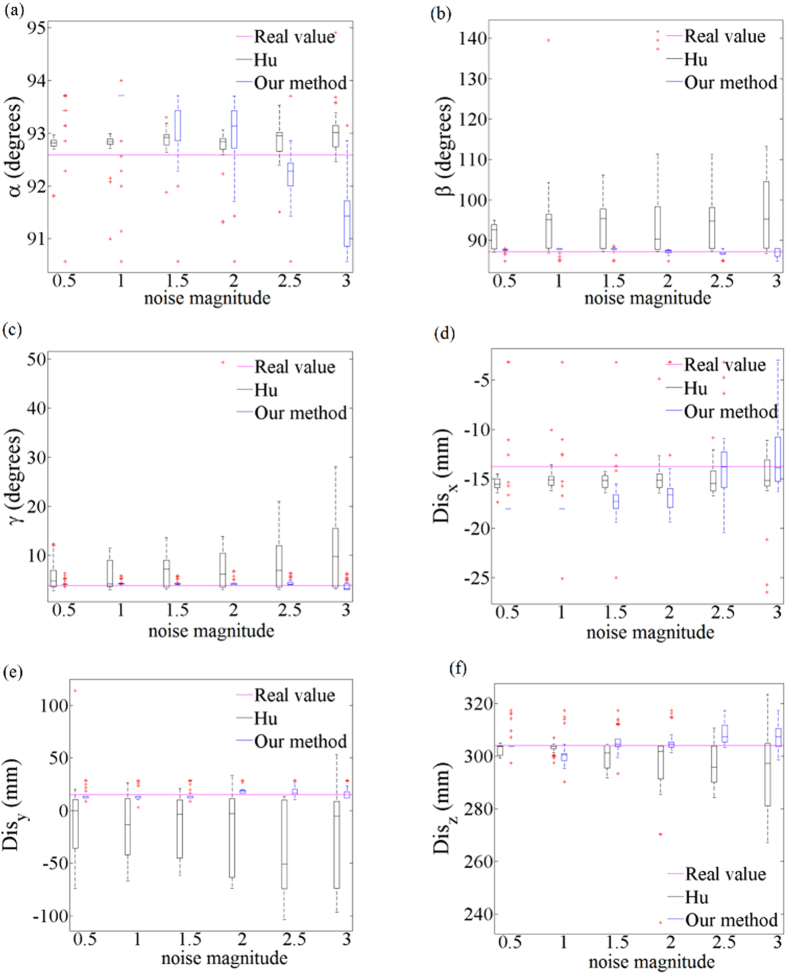
The analysis of noise influence on the three direction angles and three magnitude components of the plane normal vector in Hu’s method and our method. The horizontal axis refers to the noise magnitude with increasing amounts. The vertical axis refers to the measured three direction angles and three magnitude components of the normal vector respectively. (**a**) The relationship between the noise magnitude and the direction angle *α*. (**b**) The relationship between the noise magnitude and the direction angle *β*. (**c**) The relationship between the noise magnitude and the direction angle *γ*. (**d**) The relationship between the noise magnitude and the magnitude component *Dis*_*x*_. (**e**) The relationship between the noise magnitude and the magnitude component *Dis*_*y*_. (**f**) The relationship between the noise magnitude and the magnitude component *Dis*_*z*_.

**Figure 12:**
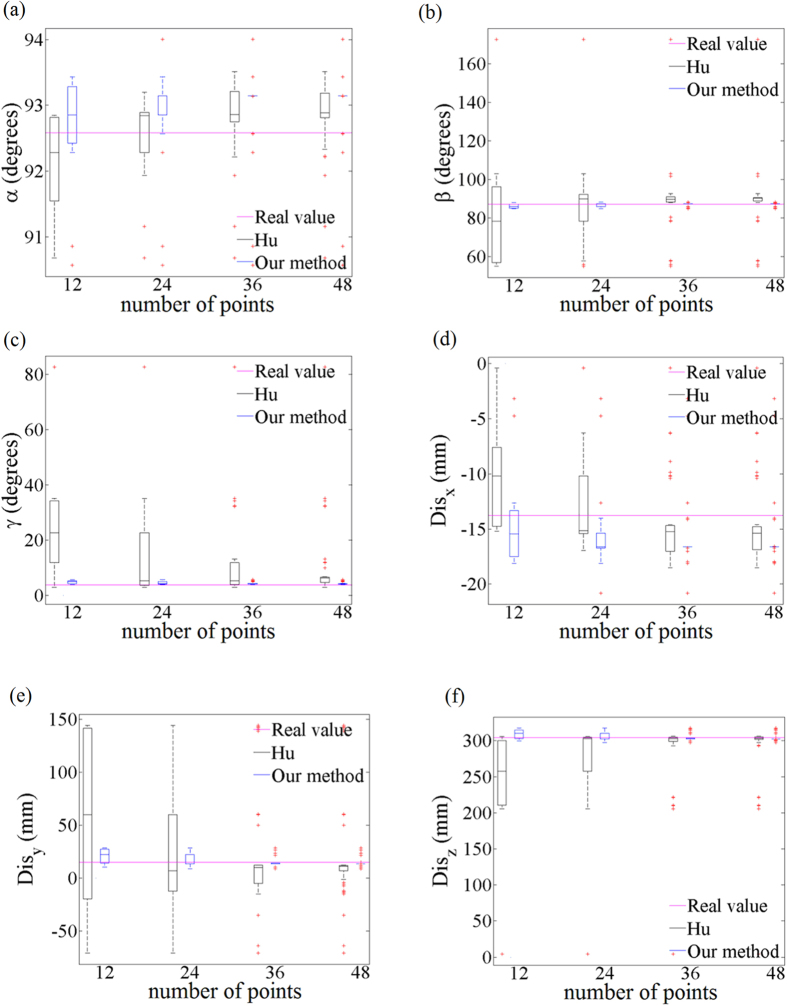
The relationship between the number of the points and the six evaluation items of the normal vector in Hu’s method and our method. The horizontal axis refers to the number of the points. The vertical axis refers to the measured three direction angles and three magnitude components of the normal vector respectively. (**a**) The relationship between the number of the points and the direction angle *α*. (**b**) The relationship between the number of the points and the direction angle *β*. (**c**) The relationship between the number of the points and the direction angle *γ*. (**d**) The relationship between the number of the points and the magnitude component *Dis*_*x*_. (**e**) The relationship between the number of the points and the magnitude component *Dis*_*y*_. (**f**) The relationship between the number of the points and the magnitude component *Dis*_*z*_.

As the laser range finders often have error modes that are highly non-Gaussian, the exponential-distribution noise is added to the image points of the camera that simulates the laser range finder. Gaussian noise is added to the image points of the other camera. In this experiment the laser plane calibration is carried using 48 points that are derived from the laser level. The image points are used as the calibration input after adding noise. Figure 11 shows the results of the three direction angles and three magnitude components of the plane normal vector, for increasing noise of the camera images. Compact result distributions and a few outliers show that the calibration method is stable. The error increases with the increasing amounts of noise smoothly. In most situations of *β*, *γ*, *Dis*_*y*_, *Dis*_*z*_, the data of our method are more concentrative than the other. The median results of *β*, *γ*, *Dis*_*y*_, *Dis*_*z*_ also approach to the real values of our method. The results of *α* and *Dis*_*x*_ are more sensitive with the increasing noise. Figure 12 shows the relationship between the number of the points and the six evaluation items of the normal vector. The 48 points are divided in 4 groups. The data of *α*, *β*, *γ*, *Dis*_*x*_, *Dis*_*y*_, *Dis*_*z*_ in our method are converging with the increase of the number of points. The data are concentrated in a smaller range and have a few outliers. The median results of *β*, *γ*, *Dis*_*y*_, *Dis*_*z*_ in our method are closer to the real values, which indicates the stability and accuracy of our method.

## Conclusions

In this work we proposed a method for a convenient and flexible calibration process of the laser plane with a simple 1D target. The 1D target is arbitrarily placed in the view filed of the camera. The bottom of the 1D target is a cone. The apex of the cone coincides with the origin of the 3D calibration board. The top of the 1D rod is marked by a checkered pattern. The laser plane passes through the center of checkered pattern on the top of the target. A group of feature points are obtained from the intersection points between the laser plane and the centers on the tops of the 1D target on different positions. A group of 2D coordinates of the intersection points are extracted from the images with the 1D target at different positions. The optimal objective function is constructed on the condition that the distance from the feature point to the the origin point of the 3D calibration board is identical to the length of the 1D target. Two optimal objective functions are acquired from the experiments, respectively. In the first case, when the equation coefficient of the laser plane *D* = −184, the first optimal objective function *f*_1_(*A*, *B*, *D*) reaches its minimal value of 1.130×10^3^. The coefficients *A*, *B* are in the scope of 0.24 < *A* < 0.26, −0.59 < *B* < −0.56. This result is consistent with the optimized coefficients *A* = 0.245, *B* = −0.575, *D* = −184 of the laser plane equation. In the second experiment, when *D* = −113, *f*_2_ (*A*, *B*, *D*) gains its minimal value of 749.249 in the scope of −0.74 < *A* < −0.71, −0.08 < *B* < −0.06. This result agrees with the calculated coefficients *A* = −0.725, *B* = −0.07, *D* = −113 of the laser plane equation. Furthermore, we project the two equations of the laser planes to *xoz* plane and *yoz* plane of the 3D calibration board. The derived projective lines overlap the real intersection lines between the laser plane and the 3D calibration board. Moreover, we observe the value of the objective function varies with the change of the group number *n* of the experimental images and the laser plane coefficient *D*. The results of these two experiments indicate that the optimal objective function inclines to the stable optimal solutions when the number of data group is close to 20 in the experiment. Finally, we add Gaussian noise to the experimental images to analyze the influence of the noise on the experiment results. The relative errors of the laser plane coefficients *A* and *B* reduce when Gaussian noise weakens and gradually diminish when the data group number *n* in the experiment increases from 0 to 20. The comparison work about the influences of the non-Gaussian noise and point number is also investigated in the experiments.The experiments demonstrate that the proposed method with the distance optimal object from the feature point to the origin point has the potential for enhancing the accuracy and robustness of the laser plane calibration in structured light measurement.

## Additional Information

**How to cite this article**: Xu, G. *et al.* An optimization solution of a laser plane in vision measurement with the distance object between global origin and calibration points. *Sci. Rep.* **5**, 11928; doi: 10.1038/srep11928 (2015).

## Figures and Tables

**Table 1:** Transformation matrices in two experiments.

	***m***_**11**_	***m***_**12**_	***m***_**13**_	***m***_**14**_	***m***_**21**_	***m***_**22**_	***m***_**23**_	***m***_**24**_	***m***_**31**_	***m***_**32**_	***m***_**33**_	***m***_**34**_
1	−1.24	0.529	−0.306	8.76 × 10^2^	1.41 × 10^-2^	5.22 × 10^-2^	−1.32	7.63 × 10^2^	−5.03 × 10^-4^	−3.99 × 10^−4^	−3.44 × 10^−4^	1
2	−1.22	0.553	−0.362	8.85 × 10^2^	5.37×10^-2^	3.10 × 10^-3^	−1.32	7.60 × 10^2^	5.11 × 10^-4^	3.43 × 10^−4^	3.73 × 10^−4^	1
